# Mating system evolution and genetic structure of diploid sexual populations of *Cyrtomium falcatum* in Japan

**DOI:** 10.1038/s41598-021-82731-1

**Published:** 2021-02-04

**Authors:** Ryosuke Imai, Yoshiaki Tsuda, Atsushi Ebihara, Sadamu Matsumoto, Ayumi Tezuka, Atsushi J. Nagano, Ryo Ootsuki, Yasuyuki Watano

**Affiliations:** 1 Sugadaira Research Station, Mountain Science Center, University of Tsukuba, Sugadaira, Ueda, Nagano 386-2204 Japan; 2 Department of Botany, National Museum of Nature and Science, Tsukuba, Ibaraki 305-0005 Japan; 3 Faculty of Agriculture, Ryukoku University, Otsu, Shiga 520-2194 Japan; 4 Department of Natural Sciences, Faculty of Arts and Sciences, Komazawa University, 1-23-1 Komazawa, Setagaya-ku, Tokyo, 154-8525 Japan; 5 Department of Biology, Graduate School of Science, Chiba University, Inage, Chiba, Chiba 263-8522 Japan

**Keywords:** Evolutionary genetics, Population genetics, Plant ecology, Plant evolution, Plant genetics, Plant reproduction, Evolutionary biology, Genetic markers, Genotype, Inbreeding, Plant breeding, Plant genetics

## Abstract

Evolution of mating systems has become one of the most important research areas in evolutionary biology. *Cyrtomium falcatum* is a homosporous fern species native to eastern Asia. Two subspecies belonging to a sexual diploid race of *C. falcatum* are recognized: subsp. *littorale* and subsp. *australe*. Subspecies *littorale* shows intermediate selfing rates, while subsp. *australe* is an obligate outcrosser. We aimed to evaluate the process of mating system evolution and divergence for the two subspecies using restriction site associated DNA sequencing (RAD-seq). The results showed that subsp. *littorale* had lower genetic diversity and stronger genetic drift than subsp. *australe*. Fluctuations in the effective population size over time were evaluated by extended Bayesian skyline plot and Stairway plot analyses, both of which revealed a severe population bottleneck about 20,000 years ago in subsp. *littorale*. This bottleneck and the subsequent range expansion after the LGM appear to have played an important role in the divergence of the two subspecies and the evolution of selfing in subsp. *littorale*. These results shed new light on the relationship between mating system evolution and past demographic change in fern species.

## Introduction

The evolution of mating systems has long been one of the most important areas of study in evolutionary biology. Transition from outcrossing to selfing is a common evolutionary pathway in angiosperms^1,2^, and selfing is considered to be advantageous for gene transmission and reproductive assurance in the short term. If repeated selfing is effective at purging genetic load, predominant selfing may constitute a stable state when inbreeding depression levels are low^3,4^. Theoretical studies predict that a population bottleneck favors selfing because it can reduce inbreeding depression^3^. Empirical studies also suggest a close relationship between bottlenecks and selfing. For example, in *Capsella* (Brassicaceae), the self-compatible *C. rubella* separated from the self-incompatible *C. grandiflora* in the last glacial period, and this speciation was associated with a major population bottleneck in *C. rubella*^5^. The selfing species *C. rubella* then expanded its distribution into new northern habitats that may have emerged after the Last Glacial Maximum (LGM)^5,6^. Since selfing would confer an advantage when filling such new habitats, the environmental shifts that occurred during the last glacial period and as it ended are considered to have favored the evolution of selfing. However, the general patterns of mating system evolution and their relationship to the demographic history of species have not yet been closely examined.

To deepen our understanding of the relationship between selfing evolution and demographic history (e.g. the occurrence of population bottlenecks), in this study we focused on a homosporous fern species, *Cyrtomium falcatum* (L.f.) C.Presl. Since self-incompatibility systems such as those based on S-loci are not observed in ferns or other seedless land plants, their mating system would be expected to be determined simply by the balance between inbreeding depression and the advantages of selfing, both of which could be affected by demographic history. The life cycle of homosporous ferns differs from that of seed plants as they have free-living gametophytes that potentially bear both male and female gametangia. The gametophytes are capable of three types of mating: gametophytic selfing (selfing within a gametophyte), sporophytic selfing (crossing between two gametophytes of the same sporophyte) and sporophytic outcrossing (crossing between two gametophytes from separate sporophytes)^7^. Among land plants, gametophytic selfing is unique to homosporous ferns and lycophytes and monoecious bryophytes, and it is considered to be an extreme form of inbreeding because it results in a zygote that is homozygous at every gene locus. Sporophytic selfing is equivalent to selfing in seed plants. These two types of selfing are responsible for inbreeding in homosporous ferns. Elucidating the evolutionary patterns and process of selfing in homosporous ferns and comparing them to those in seed plants is crucial for understanding the fundamental processes of mating system evolution in vascular plants.

*Cyrtomium falcatum* is a homosporous fern in the family Dryopteridaceae, and is native to Japan, Korea, South China, Taiwan, and Indochina. Two cytotypes have been identified: sexual diploid and apogamous triploid^8^. Matsumoto^8^ recognized two forms of the sexual diploid cytotype from the apogamous triploid, *C. falcatum* subsp. *falcatum*. These diploid cytotypes were recently formally described as two subspecies: *C. falcatum* subsp. *littorale* S.Matsumoto ex S.Matsumoto et Ebihara and *C. falcatum* subsp. *australe* S.Matsumoto ex S.Matsumoto et Ebihara^9^. *Cyrtomium falcatum* subsp *littorale* is distinguished by its smaller plant size, fewer numbers of pinnae, and the indusium does not have a black spot, cf. *C. falcatum* subsp. *australe*. Subspecies *littorale* is distributed in the north-eastern part of Japan and laboratory experiments have demonstrated variation in sexual expression (gametangium formation) in gametophytes among its populations^8^. Sporophytes referred to as the separate type (S-type) produce gametophytes bearing antheridia and archegonia that are separated both spatially and temporally. Conversely, sporophytes of the mixed type (M-type) produce gametophytes that bear antheridia and archegonia simultaneously in a mixed manner^8^. Variation in the sexual expression of gametophytes can profoundly affect the rate of gametophyte selfing in natural populations^10^. Our recent study using microsatellites showed that levels of inbreeding (*F*_IS_) and gene diversity (*h*) differed significantly between S-type (*F*_IS_ = 0.208, *h* = 0.367) and M-type (*F*_IS_ = 0.626, *h* = 0.152) populations, suggesting higher selfing rates and lower genetic diversities in M-type populations^11^. Subspecies *australe* is distributed in the southern part of Japan and exhibited little selfing ability when its gametophytes were cultured in isolation^8^. Only S-type sexual expression has been observed to date in subsp. *australe*^8^. Subspecies *littorale* may be a lineage derived from subsp. *australe* because the former subspecies has specialized ecological and morphological characters, including saxicolous habitat, early maturation, progenesis, white indusium without a central black spot, and considerable levels of selfing. The variation in the mating systems of the derived subspecies and the predominance of outcrossing in its ancestral subspecies constitute an evolutionary pattern that makes *C. falcatum* a useful model for studying mating system evolution in homosporous ferns.

In this work, restriction-site associated DNA Sequencing (RAD-seq)^12^ was performed to acquire a large amount of genetic data for high resolution population genetic analysis in order to evaluate phylogenetic relationships among S-type and M-type populations of *C. falcatum* subsp. *littorale* and S-type populations of subsp. *australe*. RAD-seq data also enabled us to estimate the demographic history of subsp. *littorale* and subsp. *australe*. In non-model organisms that are lacking extensive molecular marker development, RAD-seq is a valuable tool for molecular population genetics. We addressed two questions by means of these analyses: (1) Are S-type populations in subsp. *littorale* more closely related to subsp. *australe* than M-type ones? (2) Are there any associations between demographic events, lineage divergence and the evolution of selfing? By answering these questions, we aimed to clarify the process of mating system evolution in the two subspecies of diploid sexual *C. falcatum*.

## Materials and methods

### Preparation of samples

**Figure 1:**
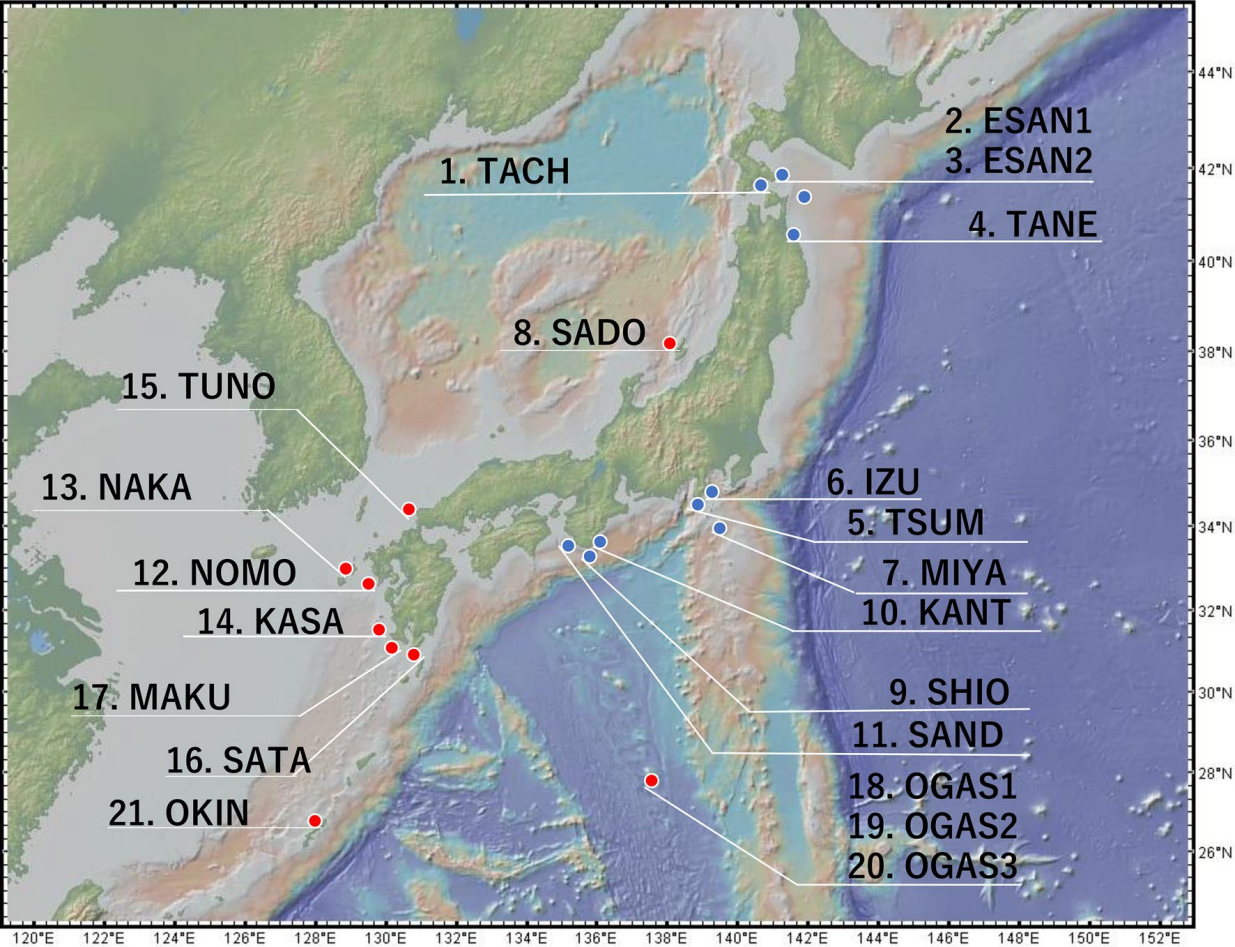
Sampling points of *C. falcatum* subsp. *australe* and *littorale* populations. The red dot shows the location of *C. falcatum* subsp. *australe* population and the blue dot shows *C. falcatum* subsp. *littorale* population. Population #8 (SADO) was originally considered as subsp. *littorale* based on the distribution information of the two subspecies in Japan^8^, but subsequent analyses suggested that this population belongs to subsp. *Austral*. The map was produced by GeoMapApp (http://www.geomapapp.org/).

Eighty-four samples of *Cyrtomium falcatum* were collected from 21 populations in Japan (Table 1, Fig. 1). Populations 1 to 11 (49 individuals) were located in the distribution range of *C. falcatum* subsp. *littorale*, while the remaining ten populations (34 individuals) were in that of *C. falcatum* subsp. *australe*^8,9^. Of the subsp. *littorale* populations, Pop6 and Pop8 are S-type and the rest are M-type^8,11^. Populations 2, 3, 6, 8 and 11 are the same as those examined in previous microsatellite analyses^11^; they were referred to as ESAN1, ESAN2, IZU2, SADO and SAND, respectively, in the previous study^11^. Most of the samples of these populations were collected during the previous work^11^, but new samples of Pop6 were collected during this study. The remaining samples were collected from natural populations or obtained from plants cultivated in Tsukuba Botanical Gardens, which were originally collected as described by Matsumoto (2003)^8^. In addition to the sporophyte samples, twelve haploid gametophyte samples were used. The gametophytes were grown from spores of a hybrid between subsp. *littorale* and *australe* (A1-55 × A2-2), which was made through artificial crossing by Matsumoto^8^. The sequence data from these haploid samples were used to check and remove contigs containing paralogous sequences. All of the 95 samples referred to above were subjected to RAD-seq. Details of the sources of the samples are shown in Table S1.

**Table 1:** Sampling location and gene diversity of *C. falcatum* subsp. *australe* and subsp *littorale* populations.

Population ID	Population name	Subspecies	*N*	Latitude	Longitude	*h*	*F*_IS_ (S.D.)^#^	*P*	*N*e
Pop1	TACH	*littorale*	1	41.745 N	140.721E	–	na	0.016	–
Pop2	ESAN1	*littorale*	6	41.811 N	141.184E	0.019	0.427 (0.014)	0.055	2.5
Pop3	ESAN2	*littorale*	5	41.812 N	141.184E	0.018	0.053 (0.013)	0.045	–
Pop4	TANE	*littorale*	2	40.509 N	141.610E	0.125	na	0.127	–
Pop5	TSUN	*littorale*	1	34.658 N	138.987E	–	na	0.009	–
Pop6	IZU	*littorale*	15	34.882 N	139.132E	0.088	0.233 (0.008)	0.288	16.7
Pop7	MIYA	*littorale*	1	34.053 N	139.482E	–	na	0.061	–
Pop8	SADO	australe*	7	38.093 N	138.250E	0.267	− 0.143(0.011)	0.678	1.6
Pop9	SHIO	*littorale*	2	33.437 N	135.754E	0.124	na	0.127	–
Pop10	KANT	*littorale*	1	33.600 N	135.600E	–	na	0.01	–
Pop11	SAND	*littorale*, australe*^,#^	8, 2	33.666 N	135.336E	0.138, ***	0.295 (0.015), na	0.642, 0.406	7.0, –
Pop12	NOMO	*australe*	4	32.594 N	129.762E	0.208	na	0.573	–
Pop13	NAKA	*australe*	8	32.921 N	129.006E	0.258	0.121 (0.009)	0.458	14.1
Pop14	KASA	*australe*	6	31.426 N	130.146E	0.283	− 0.189 (0.009)	0.738	2.4
Pop15	TSUN	*australe*	5	34.352 N	130.839E	0.25	− 0.423 (0.011)	0.58	16.8
Pop16	SATA	*australe*	1	30.995 N	130.662E	–	na	0.172	–
Pop17	MAKU	*australe*	1	31.251 N	130.279E	–	na	0.169	–
Pop18	OGAS1	*australe*	2	27.086 N	142.208E	0.311	na	0.356	–
Pop19	OGAS2	*australe*	2	27.104 N	142.193E	0.158	na	0.205	–
Pop20	OGAS3	*australe*	2	27.083 N	142.224E	0.164	na	0.234	–
Pop21	OKIN	*australe*	1	26.766 N	128.197E	–	na	0.137	–

*N* number of samples, *h* gene diversity, *F*_IS_ inbreeding coefficient, *P* proportion of polymorphic loci, *N*e effective population size.

*Samples of Pop8 and 11 were originally collected as subsp. *littorale* based on the distribution information of the two subspecies in Japan^8^, but subsequent analyses suggested that some of them belong to subsp. *australe*.

^#^Values of the two subspecies in Pop11 are shown separated by a comma.

*Samples of Pop8 and 11 were originally collected as subsp. *littorale* based on the distribution information of the two subspecies in Japan^8^, but subsequent analyses suggested that some of them belong to subsp. *australe*.

^#^Values of the two subspecies in Pop11 are shown separated by a comma.

### RAD-seq

Total DNA was extracted from silica-dried sporophyte leaves or from fresh gametophytes using the HEPES/CTAB method^13^. To detect genetic structure and population demographic events, we extracted genome wide SNP from samples using Double-digest RAD-seq (ddRAD-seq)^14^. The DNA library was prepared with two restriction enzymes, *Eco*RI and *Bgl*II as described by Sakaguchi et al.^15^. Fifty-one bp single-end sequencing was conducted in one lane of the Illumina HiSeq2500 (Illumina, San Diego, CA, USA) by Macrogen (Seoul, South Korea). Finally, 9.2 gigabases with 181 million reads were obtained.

### SNP discovery and filtering of the data

The raw fastq files were ascribed to individuals based on barcode sequences. These individual fastq files were mapped onto the chloroplast genome sequence of *C. falcatum* (NC_028705, GenBank)^16^ to exclude chloroplast sequences from the data. For this purpose, we used the BWA^17^ ‘aln’ and ‘samse’ functions with default settings. We extracted chloroplast SNPs using the ‘mpileup’ function of samtools 1.3.1^18^. After mapping, we extracted unmapped single end reads with samtools 1.3.1 ‘view’ function. These unmapped sequences were considered to contain no chloroplast sequences. After excluding chloroplast reads, we converted bam files to fastq files using bamtofastq in Hydra version 0.5.3^19^. Next, we created contigs using pyRAD^20^. Low quality reads (quality value Q < 33) were discarded. At the within-sample clustering stage, the minimum coverage (number of reads per sample), maximum number of heterozygous sites, and allowed number of alleles were set to 5, 5, and 2 respectively. The maximum proportion of shared heterozygote sites allowed was less than 40%. Variant Call Format (VCF) files were used for the following analysis, which included only SNPs generated by pyRAD. As the first data filtering step, we removed loci with > 20% missing data using vcftools^21^ because missing data can bias data analysis. We also removed individuals with > 30% missing data to avoid the adverse effects of low-quality DNA data. Next, we removed loci with minor allele frequencies (< 3%) from the non-chloroplast vcf file. Roesti et al. ^22^ suggested that minor alleles can be uninformative and lead to bias in genome scanning (e.g. detection of outlier loci), which is important when evaluating both selection and neutral genetic structure^23^. A minor allele frequency threshold of 3% has been widely used in recent NGS-based studies^24–26^. We also filtered out chloroplast loci with > 20% missing data in the same way. To estimate genetic structure based on chloroplast data, we used Splits tree4^27^ to construct a neighbor net with the chloroplast data.

### Detection of outlier loci

To evaluate genetic structure and demography based on neutral genetic information and to avoid bias due to outlier loci^21^, we conducted outlier loci detection. Although population-based outlier loci detection methods have been used extensively, a recent study^22^ showed that historical or on-going demographic events (e.g. gene flow, admixture, population bottlenecks, and expansions) can lead to bias in these population-based approaches. Moreover, admixture and a historical bottleneck were observed in *C. falcatum* populations in our previous study^11^. We therefore used an individual-based principal component analysis (PCA) approach to detect outlier loci. This approach was implemented using the R package pcadapt^28^ which robustly detects outlier loci under several population demographic patterns. A principal component number of K = 7 was initially employed based on the trend of the scree plot. However, the number of outlier loci was too large under these conditions. Because the slope of the scree plot began leveling out at K = 3, we instead set K to 3. We also excluded non-neutral loci estimated by pcadapt using vcftools. The remaining loci, which were considered to be neutral, were used for population genetic analysis.

### Genetic diversity within populations and population differentiation

The average gene diversity and *F*_ST_ values at each SNP locus were calculated using hierfstat^29^. To estimate the effect of differences in mating system, we also used hierfstat to compute the mean inbreeding coefficient, *F*_IS_, for populations of more than 4 individuals. According to Hedrick^30^, the *F*_IS_ value reflects the relative contribution of selfing sensu-lato (both gametophytic and sporophytic selfing) to outcrossing in each population. To validate the geographical pattern of inbreeding coefficients, we also tested the correlation between *F*_IS_ and latitude for subsp. *littorale* and *australe* populations. We estimated effective population size by linkage disequilibrium using NeEstimator 2.01^31^.

### Individual-based genetic structure and admixture

To evaluate genetic structure among individuals, we performed principal coordinate analysis (PCoA) using GenAlEx 6.5^32^. To carry out the PCoA, pairwise genetic distances^33^ between individuals were calculated with the “interpolating missing” option to correct for any bias due to missing data. We also conducted a model-based cluster analysis using STRUCTURE^34^. Numbers of clusters (*K*) between 1 and 25 were evaluated under the correlated allele frequencies model^35^ by running 10,000 burn-in Markov Chain Monte Carlo (MCMC) repetitions and 10,000 subsequent repetitions. We used the CLUMPAK server^36^ to evaluate multimodality^37^ among runs for each *K* and to generate barplots for the different values of *K*. The probability of the data (Ln P(D)) and *ΔK*^38^ were summarized using STRUCTUREHARVESTER^39^. We estimated a pattern of population bifurcation and events of mixing between populations by using TreeMix 1.12^40^. TreeMix infers the points at which admixtures happened in a population tree under the assumed number of admixture events. We assumed 1–5 admixture events, and the best tree was defined by likelihood^41^.

### Demographic inference

Population demography in terms of temporal changes in population size was evaluated by generating an Extended Bayesian Skyline Plot (EBSP)^42^ and a Stairway plot^43^. We first generated an EBSP using BEAST 2.4.4^44^ for the two subspecies, excluding the OGAS populations. We used a modified version of the method introduced by Trucchi et al*.*^45^, who successfully detected temporal changes in the size of king penguin populations and showed that highly polymorphic loci identified by RAD-seq can reflect general trends that also apply to other kinds of loci. To obtain all sequences with more than four polymorphic sites, following Trucchi et al.^45^, we sampled one sequence per individual randomly. We used a substitution rate of 0.75 × 10^–8^ per site per year based on estimates for *Arabidopsis thaliana*^46^. The substitution rate parameter for sequences with four polymorphic sites was set to 1.0, and was increased in proportion to the number of polymorphic sites (i.e. it was set to 1.25 for sequences with five polymorphic sites and 1.5 for those with six). We ran 250,000,000 MCMC chains and collected a trace and EBSP log after every 1000 chains. Tracer 1.6^47^ was used to verify that the Estimated Sample Size was greater than 100 for all parameters. We calculated Tajima’s D^48^ for these loci using DnaSP 6^49^ to evaluate the general trend in the demographic pattern.

Stairway plot v2^43^ is a newly developed method for inferring temporal changes in population size by using site frequency spectrum (SFS) data to test demography. To estimate SFS, we used a sequence from the individual in Pop 17 as the ancestral sequence because the TreeMix results indicated that this population was at the base of the population tree. We mapped sequence data for all individuals onto this sequence using BWA^17^ and used samtools 1.8^18^ to convert SAM files to BAM files and sort the BAM files. We estimated the site frequency spectrum with ANGSD^50^, using the following options: dosaf 1 (multi sample genotype likelihood estimation with assumed Hardy–Weinberg equilibrium), GL: 1 (genotype likelihood estimation of the samtools model), and RealSFS using the maximum number of iterations (100). We used the same mutation rate as for EBSP and assumed a generation time of three years in the demography estimation for the Stairway plot.

## Results

### Data filtering

The non-chloroplast dataset contained 9350 loci before filtering. After excluding low frequency minor alleles and missing data, 2636 loci remained. Pcadapt detected 355 loci estimated to be non-neutral among these loci. Thus, 2281 loci were used for population genetic analyses. For chloroplast DNA (cpDNA), 191 SNPs were extracted and 180 remained after excluding missing data. Although 10 haplotypes were detected, most of the individuals were fixed for haplotype 1 (Table S1) and the neighbor net (Fig. S1) showed no clear pattern. Thus, no clear genetic structure was detected at the cpDNA level.

### Genetic diversity, structure and admixture

Gene diversity was low in subsp. *littorale*. Subspecies *australe* had higher gene diversity than all the subsp. *littorale* populations except for Pop8 (SADO). SADO had the highest gene diversity of the subsp. *littorale* populations, but subsequent analyses suggested that this population belongs to subsp. *australe* (see the STRUCTURE and TreeMix analysis below). Taking this result into account, all subsp. *australe* populations had higher gene diversity than subsp. *littorale* (Table 1). The *F*_ST_ value over all populations was 0.424. The *F*_ST_ value between the two subspecies was 0.357 when the SADO population was treated as subsp. *australe*. The inbreeding coefficient *F*_IS_ was higher in subsp. *littorale* populations than subsp. *australe* populations, some of which showed negative *F*_IS_ values.

**Figure 2:**
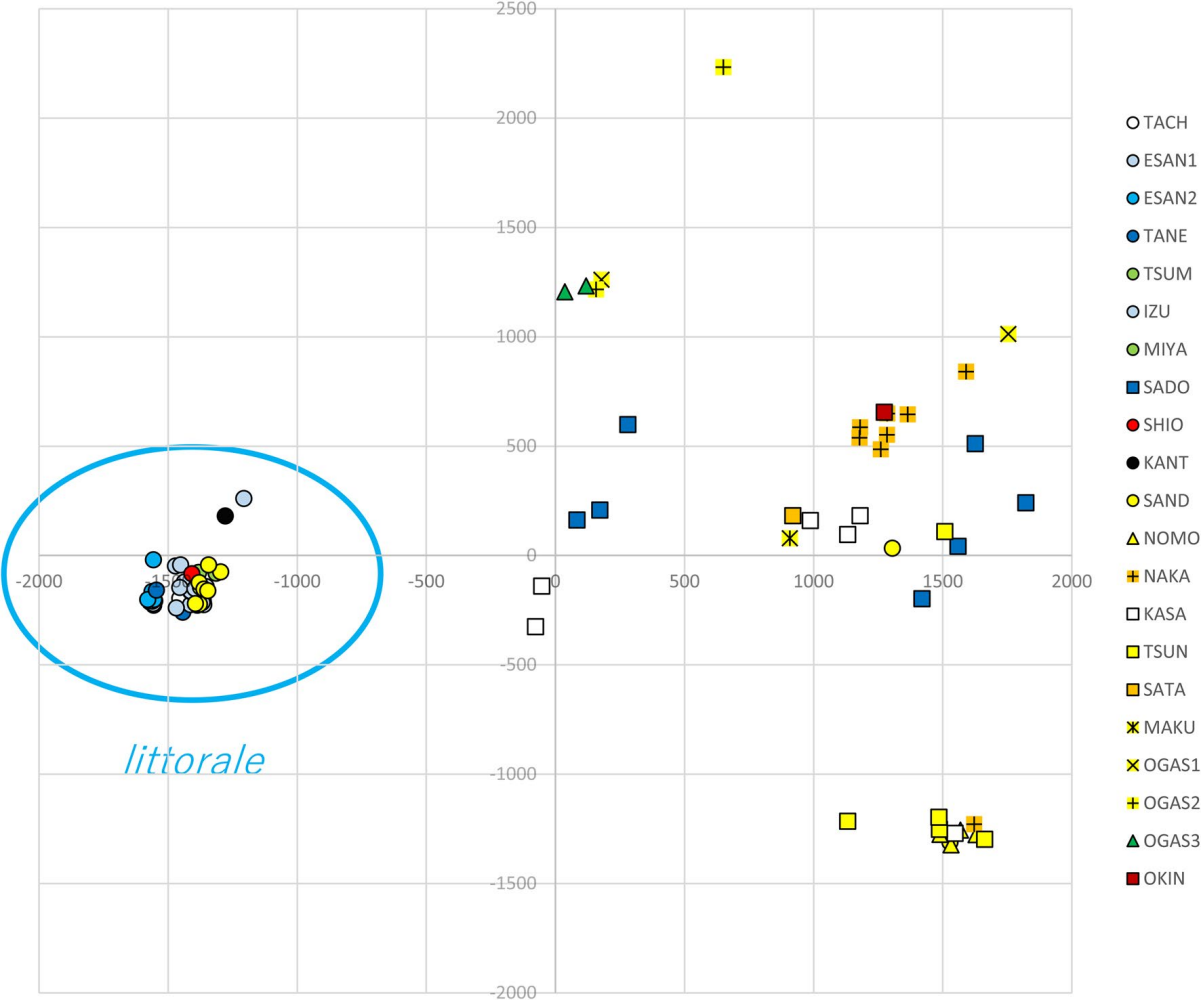
A principle component analysis of *C. falcatum* subsp. *australe* and *littorale* individuals. *Subspecies littorale* individuals were grouped within a narrow range, while subsp. *australe* did not show clear clustering.

PCA showed that subsp. *littorale* individuals generally clustered together in a narrow range, whereas SADO individuals were clustered with subsp. *australe* or located between subsp. *littorale* and *australe* (Fig. 2). Two individuals in the SAND population were also within the cluster of subsp. *australe*; one of these individuals had been detected as an admixed individual in our previous study^11^. Subspecies *australe* showed greater variance and several populations were divided into multiple clusters.

In the STRUCTURE analysis of all populations, the probability of the data (LnP(D)) increased progressively with each K until it reached a plateau at *K* = 6 (Fig. S2). The highest Δ*K* was detected when *K* = 2 (Fig S3). At *K* = 2, the clustering mostly supported taxonomical classification of subsp. *littorale* and *australe*; however, all of the SADO and two of the SAND individuals were included in the *australe* cluster or showed an admixture-like pattern. The OGAS and KASA populations also showed population admixture at *K* = 2, *K* = 3, and *K* = 4. However, at *K* = 5 the OGAS1—3 populations formed a new cluster. At *K* = 3, several subsp. *australe* populations appeared as admixtures of multiple clusters. At *K* = 4, NOMO and TSUN appeared as pure clusters; this outcome was observed up to *K* = 25.

**Figure 3:**
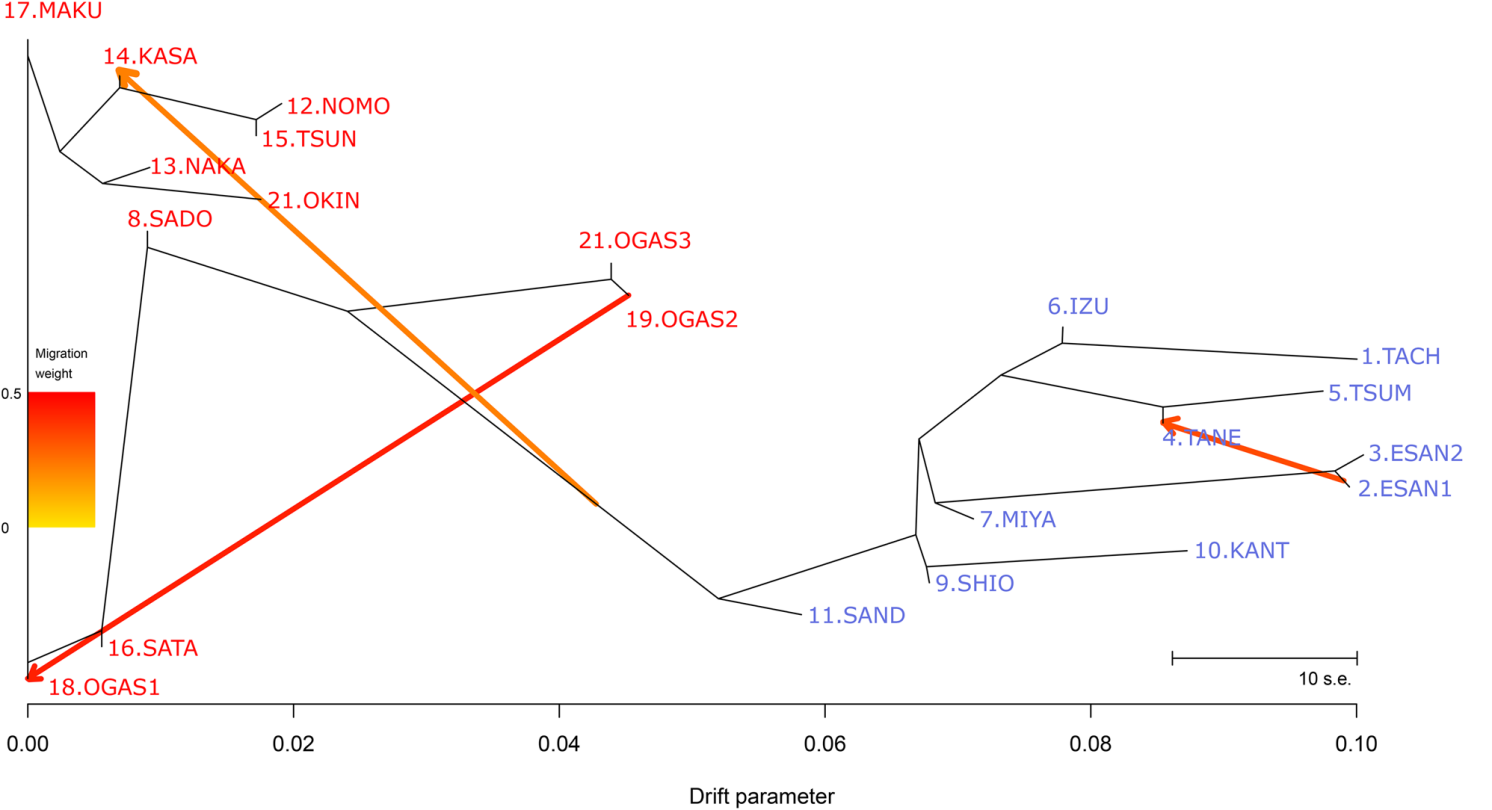
A population tree of *C. falcatum* subsp. *australe* and subsp. *littorale* populations using TreeMix. The x-axis is the drift parameter from the assumed common ancestral population. The orange arrows are strength and direction of gene flow. The scale bar indicates ten times the standard error.

The TreeMix analysis showed that the likelihood increased with the assumed number of migration events. We identified subsp. *australe* as the ancestral form on the basis of gene diversity, so we presumed that subsp. *australe* would have a lower drift parameter. This was the case when the migration number *m* was set to 3. All subsp. *littorale* populations other than the SADO population constituted one group that departed from subsp. *australe* (Fig. 3). TreeMix showed a pattern of divergence from south to north in subsp. *littorale*; three populations on the Kii Peninsula (SAND, SHIO, and KANT) near the western limit of the subspecies’ distribution (Fig. 1) were located at the base of the clade of subsp. *littorale*. S-type (low selfing rate) populations in subsp. *littorale* were placed in two different positions of the tree: SADO was in subsp. *australe*, and IZU was in a branch of subsp. *littorale*. The populations from the Ogasawara Islands were divided into two groups: OGAS1 had a low drift parameter, while OGAS2 and 3 formed a group sister to subsp. *littorale* (Fig. 3).

### Demographic inference and temporal changes in effective population size

**Figure 4:**
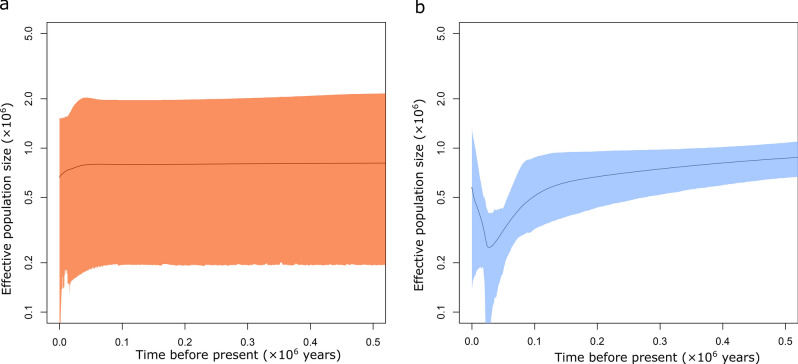
Effective population size of *C. falcatum* subsp. *australe* **(a)** and subsp. *littorale* **(b)** in chronological order estimated by Extended Bayesian Skyline Plot. The x-axis is time in million years before present. The y-axis is the effective population size in million individuals. The 95% confidence interval is filled red **(a)** and blue **(b)**.

An EBSP analysis was performed using 82 loci for *littorale* and 77 loci for *australe*. This indicated that the population size of subspecies *littorale* fell between 100,000 and 22,000 years before the present (BP) and then increased from 22,000 years BP onwards (Fig. 4b). The effective population size at 22,000 years BP was estimated to be around 250,000 individuals based on the assumption of three years per generation. Although the confidence interval was wide, the general pattern still applied even when the width of this interval was taken into account. Conversely, subsp. *australe* showed an almost constant population size (Fig. 4a). The mean values of Tajima’s *D* of the loci examined for subsp. *littorale* and *australe* were 0.151 (SD: 1.109) and -0.025 (SD: 1.080), respectively.

**Figure 5:**
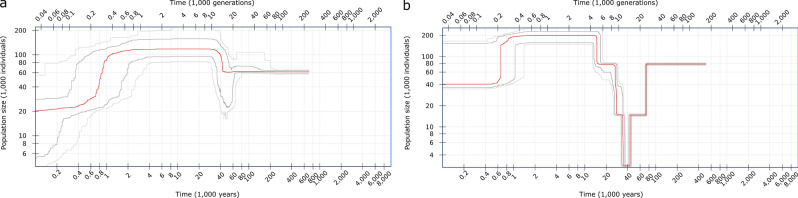
Effective population size of *C. falcatum* subsp. *australe* **(a)** and subsp. *littorale* **(b)** in chronological order estimated by Stairway plot. The x-axis is the time in thousand years (lower scale bar) and in thousand generations (upper scale bar) before present. The y-axis is the effective population size in thousand individuals. The red line shows median of effective population size. The upper and lower thick and light gray lines show 75% and 95% confidence intervals, respectively.

The Stairway plot also revealed a population bottleneck in subsp. *littorale*; the effective population size fell from about 7000 years BP and reached its minimum at 40,000 years BP (Fig. 5b). The population size began to increase after the bottleneck but decreased again 600 years BP; the effective current population size was found to be about 40,000 individuals. The *australe* subspecies exhibited no population bottleneck (Fig. 5a); its estimated current effective population size was about 20,000 individuals. The effective population size of subsp. *littorale* before the population bottleneck was about 80,000; that of subsp. *australe* was 60,000 during the same period.

## Discussion

### Genetic structure of subspecies *littorale* and *australe*

**Figure 6:**
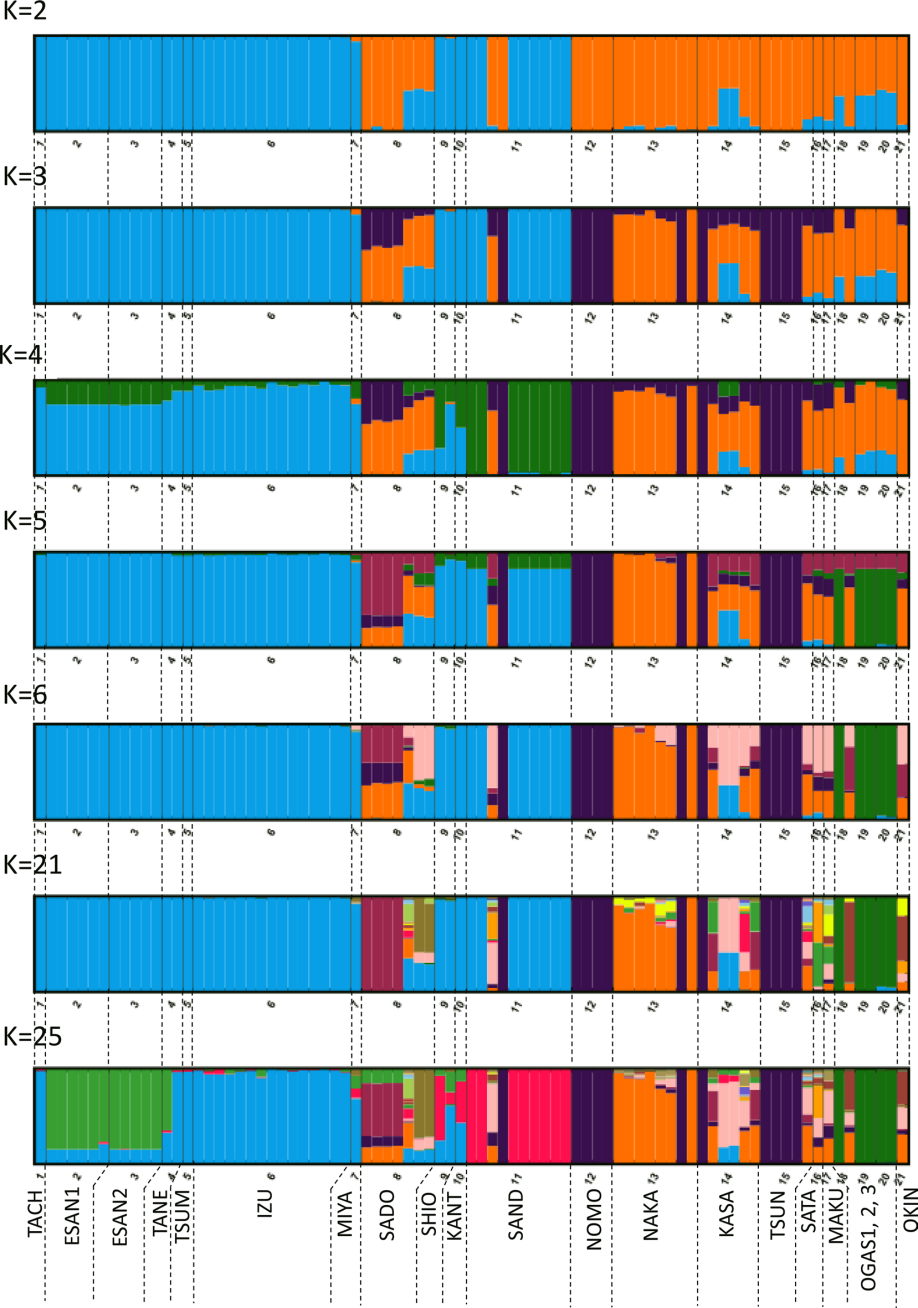
The STRUCTURE analysis of *C. falcatum* subsp. *australe* and subsp. *littorale*. The proportion of the membership coefficient of 83 individuals in the 20 populations for each of the inferred clusters for K = 2, 3, 4, 5, 6, 21 and 25 defined using Bayesian clustering in STRUCTURE.

PCA (Fig. 2) and STRUCTURE (Fig. 6) analyses showed that the SADO population and two individuals of the SAND population belong to subsp. *australe* despite its morphological characters with respect to indusium color and plant size. TreeMIX analysis (Fig. 3) also supported this conclusion. Unlike other populations of subsp. *littorale*, the SADO population had the same level of gene diversity as subsp. *australe* and showed no sign of selfing^11^ (Table 1). We therefore treated the SADO population as subsp. *australe* in this study and in the discussion below. Based on this classification, subsp. *littorale* was clearly divided from subsp. *australe* by our analyses, and was characterized by lower genetic diversity and higher selfing rates than subsp. *australe*.

Selfing reduces effective population size and results in increasing population differentiation caused by genetic drift. However, PCA revealed a low level of variance among subsp. *littorale* populations, which would not result from genetic drift by selfing. The low genetic variation of subsp. *littorale* compared to subsp. *australe* suggests that the ancestor of subsp. *littorale* experienced strong genetic drift. Neighbor net analysis based on chloroplast DNA showed no clear clustering for *littorale* and *australe* populations. This was presumably due to the small number of SNP sites in the cpDNA and the difference in substitution rate between the chloroplast and the highly polymorphic nuclear loci.

Both subsp. *australe* and subsp. *littorale* individuals were found in the SAND population (Fig. 6), which is close to the geographical boundary of the two subspecies (Fig. 1). A previous morphological study indicated that this boundary is located on the western side of Shikoku^9^. In contrast to previously reported findings^8,9^, it may be that these two subspecies do not show strictly parapatric distribution and that their distribution ranges overlap in Shikoku and on the western side of the Kii peninsula.

### Demographic history of subspecies *littorale* and *australe* populations

The two subspecies showed different demographic patterns and levels of genetic diversity. Subspecies *littorale* showed a trend indicative of population shrinkage or a bottleneck, which would result in low genetic diversity in modern populations of subsp. *littorale*. Site frequency spectrum simulations conducted during a recent study by Lapierre et al. ^51^ indicated that Stairway plots are biased by noise intrinsically present in the data when inferring true demographic history, and that caution is therefore needed when interpreting them. However, since the demographic trends indicated for subsp. *littorale* and *australe* by the Stairway plot were consistent with those determined by EBSP, we consider the general patterns of temporal change in population size presented here to be reliable. While the two methods suggested slightly different timings for the identified changes in population demography, both of them indicated that the timing of the bottleneck was related to the last glacial period. A study of *Asplenium* species in Europe demonstrated the effects of the last glacial period on genetic diversity and species range^52^. Subspecies *littorale* appears to have undergone population shrinkage during the last glacial period, whereas subsp. *australe* showed no evidence of any population shrinkage. Indeed, Tajima’s D value for subsp. *australe* was almost zero, suggesting that its effective population size was constant, neither shrinking nor expanding. The relative geographic positions of the two subspecies may explain their different demographic histories: the area providing suitable habitats for the northern subsp. *littorale* is likely to have been reduced significantly during the last glacial period, whereas that for the southern subsp. *australe* would have been largely unaffected. Alternatively, it may be that a more northerly population of this species was isolated in a refugium (for example, in the southern edge of the Kii Peninsula or Shikoku Island) and simultaneously experienced population shrinkage in the last glacial period, and that this population became a founder of subsp. *littorale*. In other words, the postulated population bottleneck event may be closely associated with the divergence between subsp. *littorale* and *australe*. Both the EBSP and Stairway plot analyses suggest that the effective population sizes for the two species were similar before the bottleneck event, which supports the second scenario.

The mixed mating system of subsp. *littorale* would have helped population size recovery after the population bottleneck. Both the EBSP and Stairway plot methods indicated a rapid increase in population size of subsp. *littorale* after the population bottleneck, which could be explained by range expansion to the north. Gametophytic selfing has been considered advantageous for reproductive assurance during rapid range expansion because it enables single-spore colonization of distant regions^53^. After the last glacial period, habitats suitable for subsp. *littorale* would have been empty in the northern area. M-type sexual expression and relatively high levels of selfing (Table 1) in subsp. *littorale* would be advantageous in filling empty habitats because they facilitate dispersal and population establishment.

### Mating system evolution in subspecies *littorale*

In our previous study using microsatellite markers, we calculated inbreeding coefficients (*F*) for the subsp. *littorale* populations to estimate their levels of selfing and obtained *F* values ranging from 0.22 to 0.79^11^. The IZU population was known to have S-type sexual expression^8^ and had the lowest *F* value (0.22). The present study using genome-wide SNP data yielded lower *F* values, ranging from 0.053 to 0.427 for the subsp. *littorale* populations (Table 1). The *F* values obtained here may be biased due to the small sample sizes of 4–15 and/or possible contamination by non-neutral loci despite multiple filtering steps. The bias appears to be towards negative *F* values, as most of the subsp. *australe* populations that seem to be outcrossers showed negative *F* values. Whereas, the IZU population, which had an adequate sample size (15) for estimating allele frequencies, had nearly the same *F* value (0.233) as that in our previous study^11^. TreeMix analysis (Fig. 3) showed a divergence pattern of populations from south to north in the clade of subsp. *littorale*, and the IZU population located in the middle of the distribution range (Fig. 1) was placed in a derived position in the tree of subsp. *littorale*. This branching order of populations is consistent with the scenario of northward distribution expansion of subsp. *littorale* after the last glacial period. However, it is not concordant with the distribution of sexual expression (S-type vs. M-type). There are two possible explanations for the non-concordance between sexual expression and tree topology. First, after the evolution of M-type sexual expression favoring selfing in the common ancestor of subsp. *littorale* populations, S-type sexual expression may have evolved newly in the IZU population or been acquired through introgression from subsp. *australe*. Second, M-type sexual expression may have emerged later on each of the *littorale* branches via parallel evolution or gene flow from the population in which M-type evolved first. In this view, IZU is a unique population that has maintained ancestral S-type sexual expression. Although IZU has S-type sexual expression like subsp. *australe*, the population shows mixed-mating (*F*_IS_ = 0.233), unlike the obligate outcrossing mating system shown by subsp. *australe* populations (Table 1). This makes the former explanation seem more likely. In both scenarios, mating system evolution must have occurred within a short period, which implies that the mating system of ferns is less stable than that of seed plants. Despite this, mating system transition is likely to be a rare event. The S-type sexual expression of subsp. *littorale* was reported in two distant locations: Sadogashima Island and Izu peninsula^8^. Because the present study excluded the SADO population on Sadogashima Island from subsp. *littorale*, only the populations around the IZU population are known to have the S-type sexual expression. Although IZU shows the lowest level of selfing among subsp. *littorale* populations, it retains a mixed mating system. Therefore, the putative transition from M-type to S-type in IZU is not an unequivocal example of recovery of outcrossing from selfing, which has not been observed in seed plants^54^. However, it can safely be described as an interesting example in which an evolutionary change that enhanced outcrossing emerged in mixed-mating plants.

The mating system of ferns seems to be determined by the balance between inbreeding depression and the advantages of selfing. Additionally, it appears that changes in the sexual expression pattern, timing, and the position of the reproductive organs on gametophytes can alter the ease of gametophytic selfing. As discussed previously^11^, the evolution of selfing via transmission advantage operates only under a low genetic load and predominant selfing is expected^3^. Therefore, the existence of mixed-mating even in the M-type populations suggests that reproductive assurance, rather than transmission advantage, is the main factor affecting the evolution of selfing in this species. If this is the case, populations of subsp. *littorale* are expected to harbor sufficient genetic load to avoid the positive feedback process of selfing evolution driven by transmission advantage^3^. If genetic diversity and inbreeding depression increased in selfing populations that have been stable for a long time as a result of migration, it would be possible for them to change their sexual expression toward enhancing outcrossing, especially in populations that were not completely selfing.

## Conclusion

This study successfully showed that *C. falcatum* subsp. *littorale* underwent a population bottleneck during the last glacial period, which appears to have induced the divergence of *C. falcatum* subsp. *littorale* and *australe.* The evolution of selfing may have been favored by reproductive assurance during rapid range expansion after the last glacial period. This is the first study to address the relationship between mating system evolution and range expansion after a glacial period based on demographic inferences, and it sheds new light on mating system evolution and ecological dynamics in both ferns and other plant species.

## Supplementary Information

Supplementary Information.
