# Author Correction: Mating system evolution and genetic structure of diploid sexual populations of *Cyrtomium falcatum* in Japan

**DOI:** 10.1038/s41598-021-98822-y

**Published:** 2021-09-21

**Authors:** Ryosuke Imai, Yoshiaki Tsuda, Atsushi Ebihara, Sadamu Matsumoto, Ayumi Tezuka, Atsushi J. Nagano, Ryo Ootsuki, Yasuyuki Watano

**Affiliations:** 1 Sugadaira Research Station, Mountain Science Center, University of Tsukuba, Sugadaira, Ueda, Nagano 386-2204 Japan; 2 Department of Botany, National Museum of Nature and Science, Tsukuba, Ibaraki 305-0005 Japan; 3 Faculty of Agriculture, Ryukoku University, Otsu, Shiga 520-2194 Japan; 4 Department of Natural Sciences, Faculty of Arts and Sciences, Komazawa University, 1-23-1 Komazawa, Setagaya-ku, Tokyo, 154-8525 Japan; 5 Department of Biology, Graduate School of Science, Chiba University, Inage, Chiba, Chiba 263-8522 Japan

Correction to: *Scientific Reports* 10.1038/s41598-021-82731-1, published online 04 February 2021

In the original version of this Article, Yasuyuki Watano was omitted as a corresponding author. Correspondence and request for materials should also be addressed to watano@faculty.chiba-u.jp. In addition, the authors Ryosuke Imai and Yoshiaki Tsuda were omitted as equally contributing authors.

The original Article and accompanying Supplementary Information file have been corrected.

