# Supplementary material for: Mating system evolution and genetic structure of diploid sexual populations of Cyrtomium falcatum in Japan
Source: Sci Rep. 2021 Feb 4;11:3124. doi: 10.1038/s41598-021-82731-1 (PMC7862634; doi:10.1038/s41598-021-82731-1)
Supplement: Supplementary file 1 — Supplementary Information. [file 41598_2021_82731_MOESM1_ESM.pptx]

## Slide 1
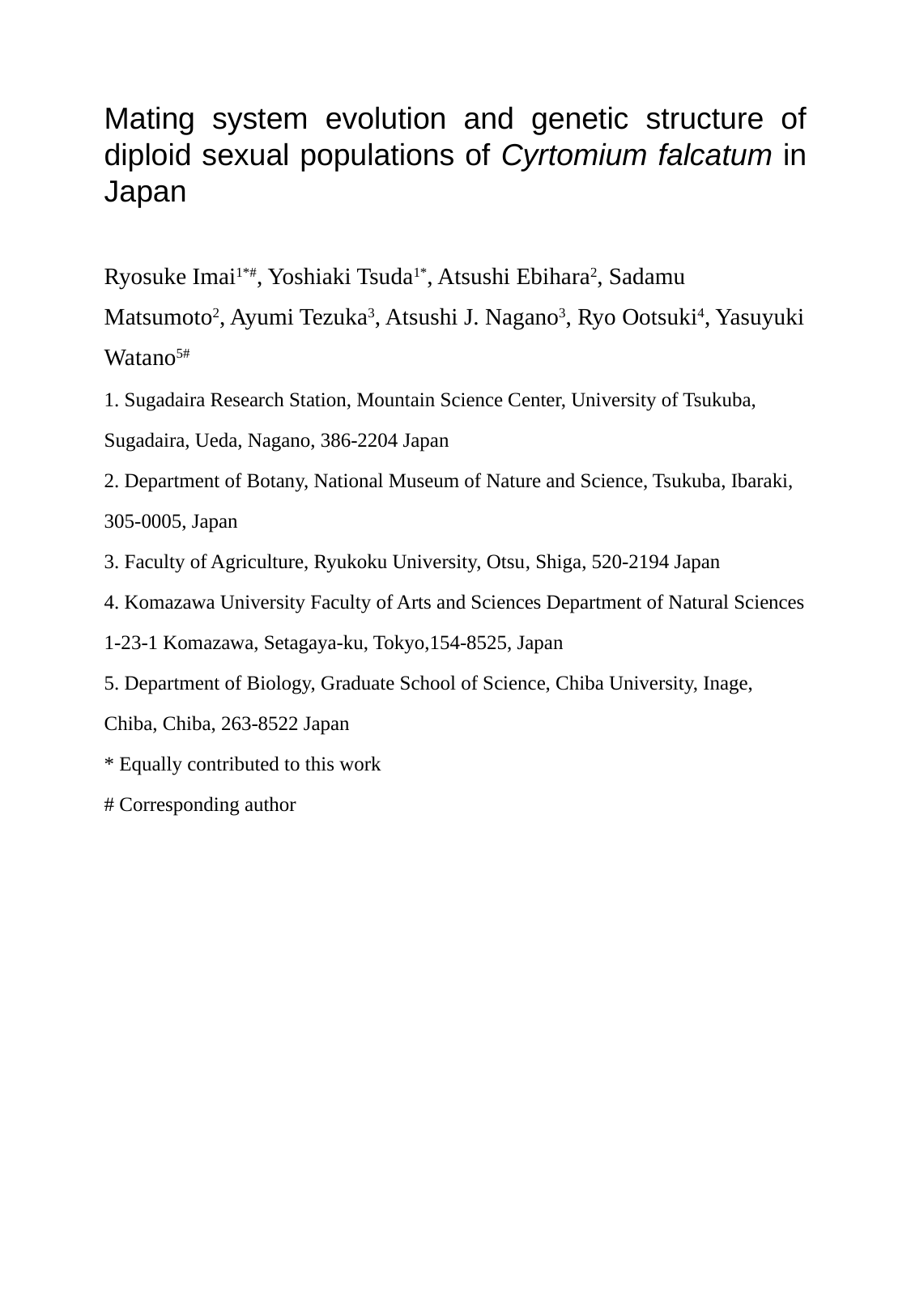

Mating system evolution and genetic structure of diploid sexual populations of Cyrtomium falcatum in Japan
Ryosuke Imai1*#, Yoshiaki Tsuda1*, Atsushi Ebihara2, Sadamu Matsumoto2, Ayumi Tezuka3, Atsushi J. Nagano3, Ryo Ootsuki4, Yasuyuki Watano5#
1. Sugadaira Research Station, Mountain Science Center, University of Tsukuba, Sugadaira, Ueda, Nagano, 386-2204 Japan
2. Department of Botany, National Museum of Nature and Science, Tsukuba, Ibaraki, 305-0005, Japan
3. Faculty of Agriculture, Ryukoku University, Otsu, Shiga, 520-2194 Japan
4. Komazawa University Faculty of Arts and Sciences Department of Natural Sciences 1-23-1 Komazawa, Setagaya-ku, Tokyo,154-8525, Japan
5. Department of Biology, Graduate School of Science, Chiba University, Inage, Chiba, Chiba, 263-8522 Japan
* Equally contributed to this work
# Corresponding author

## Slide 2
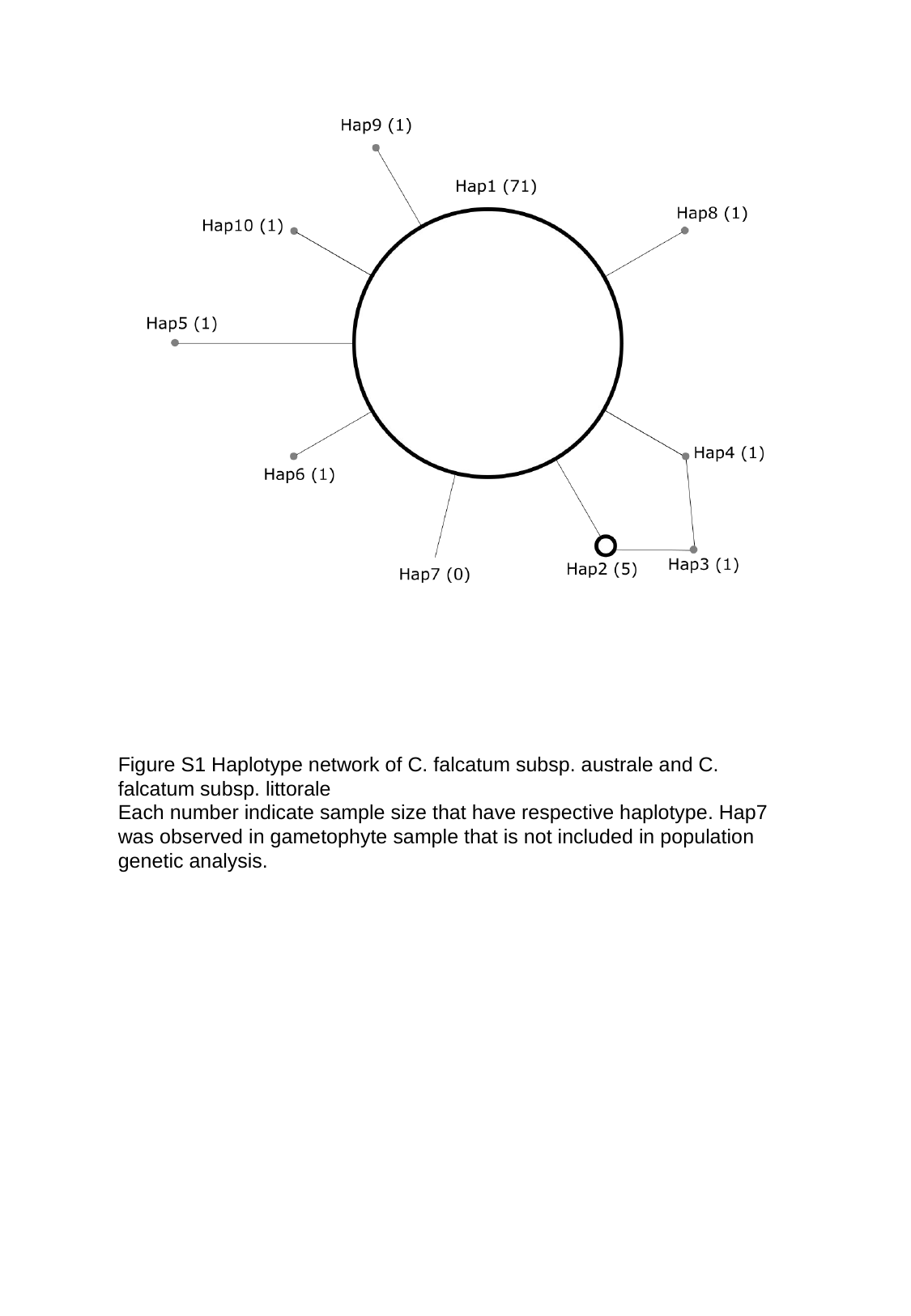

Figure S1 Haplotype network of C. falcatum subsp. australe and C. falcatum subsp. littorale
Each number indicate sample size that have respective haplotype. Hap7 was observed in gametophyte sample that is not included in population genetic analysis.

## Slide 3
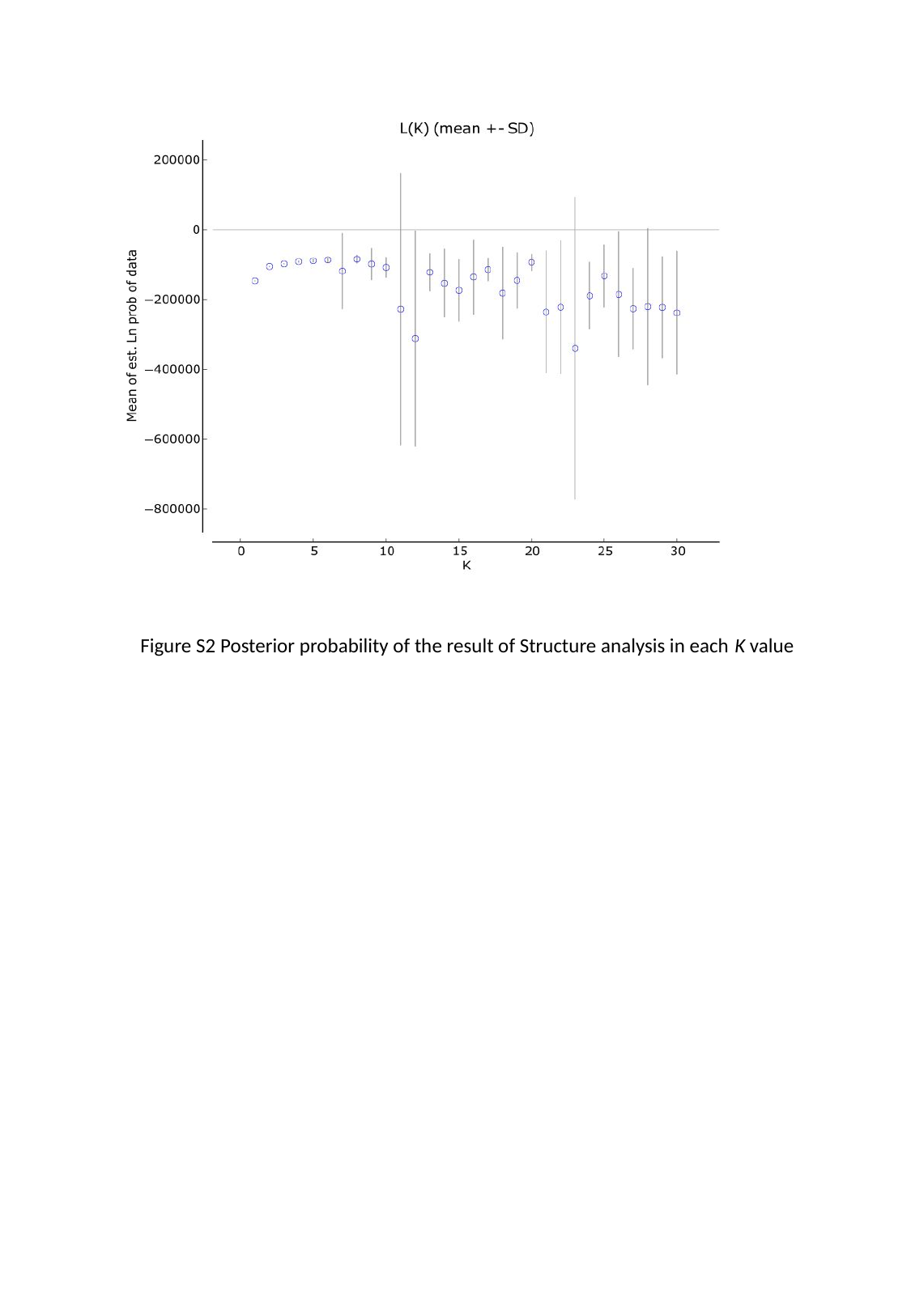

Figure S2 Posterior probability of the result of Structure analysis in each K value

## Slide 4
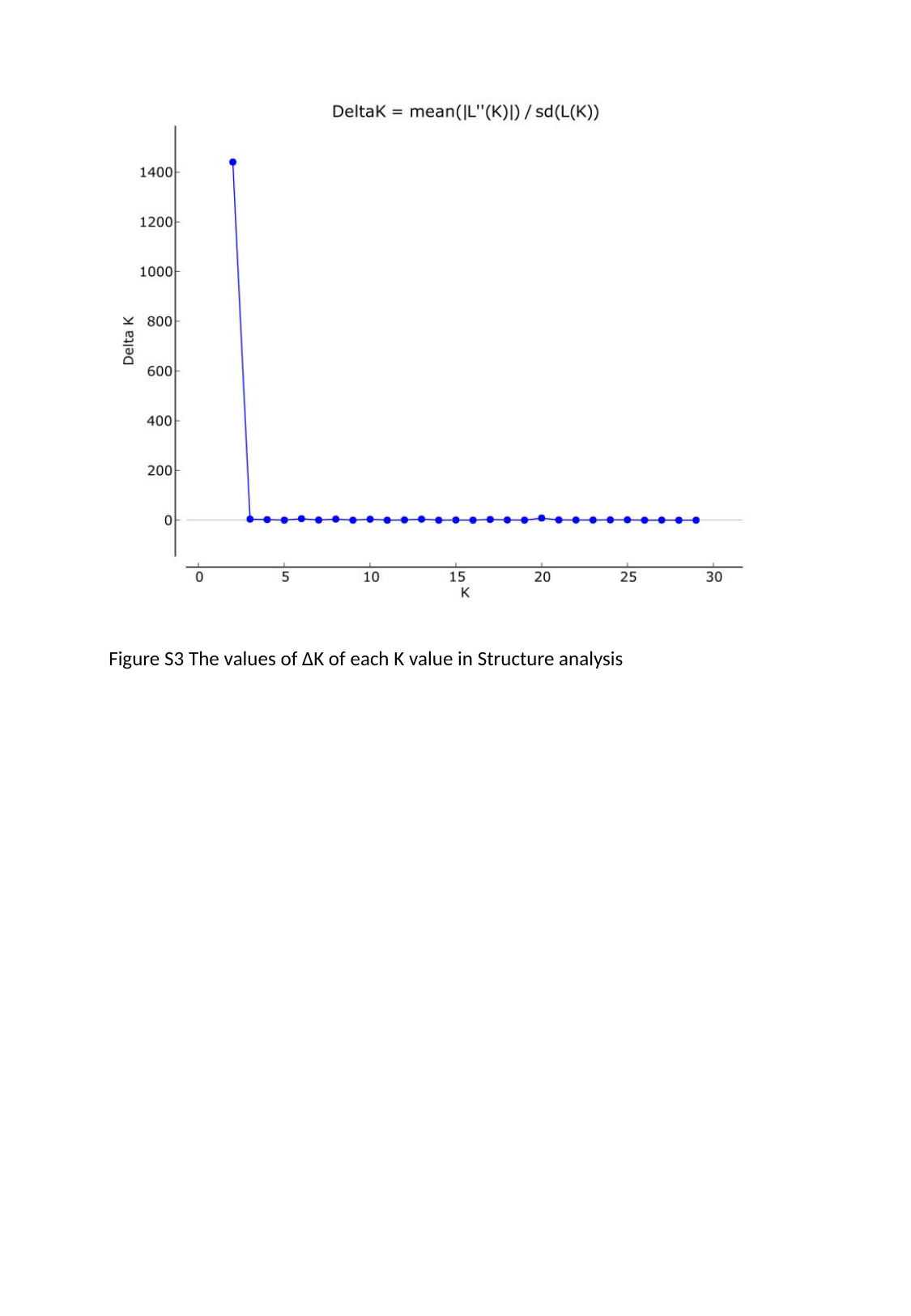

Figure S3 The values of ΔK of each K value in Structure analysis

## Slide 5
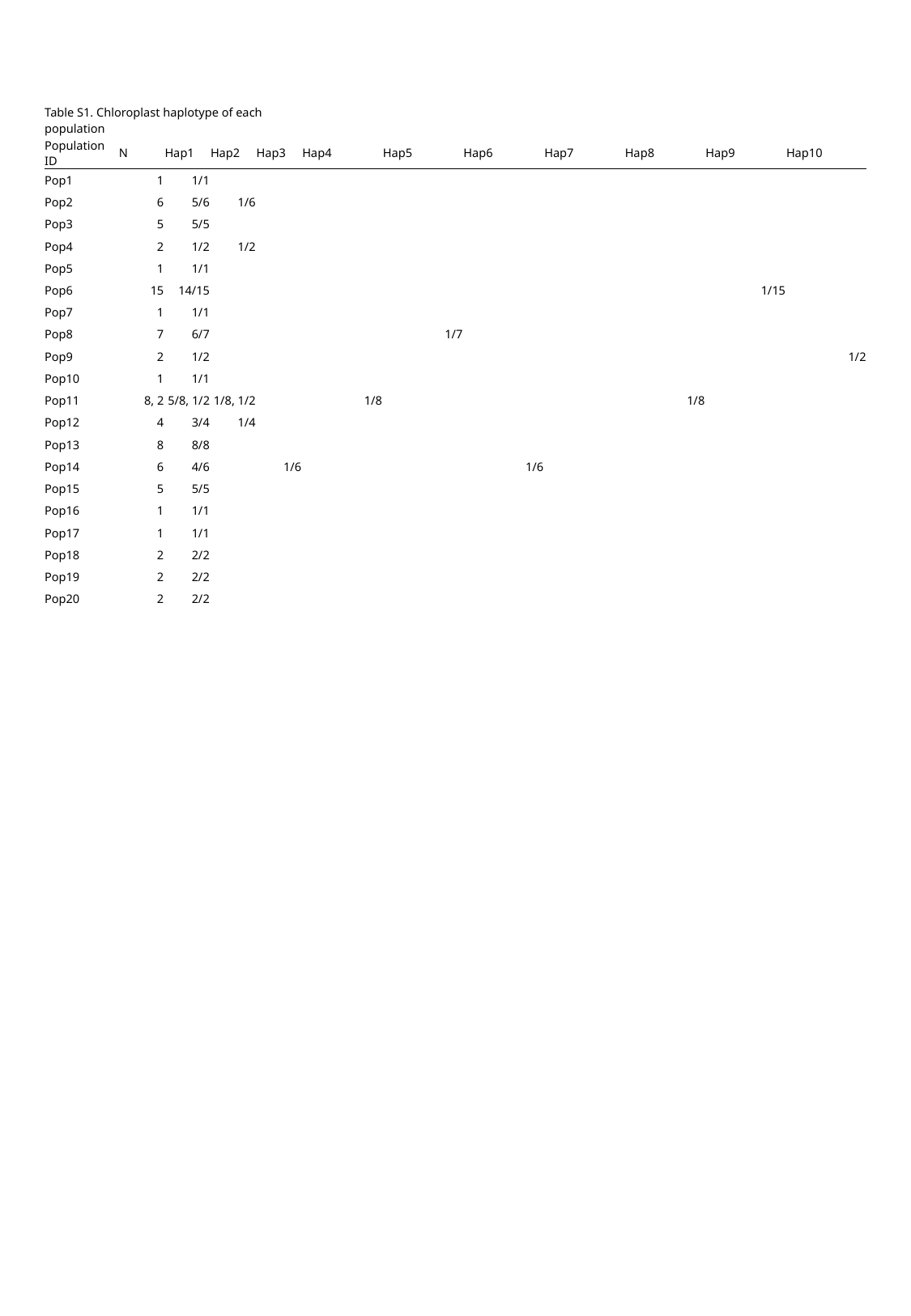

| Table S1. Chloroplast haplotype of each population | | | | | | | | | | | |
| --- | --- | --- | --- | --- | --- | --- | --- | --- | --- | --- | --- |
| Population ID | N | Hap1 | Hap2 | Hap3 | Hap4 | Hap5 | Hap6 | Hap7 | Hap8 | Hap9 | Hap10 |
| Pop1 | 1 | 1/1 | | | | | | | | | |
| Pop2 | 6 | 5/6 | 1/6 | | | | | | | | |
| Pop3 | 5 | 5/5 | | | | | | | | | |
| Pop4 | 2 | 1/2 | 1/2 | | | | | | | | |
| Pop5 | 1 | 1/1 | | | | | | | | | |
| Pop6 | 15 | 14/15 | | | | | | | | 1/15 | |
| Pop7 | 1 | 1/1 | | | | | | | | | |
| Pop8 | 7 | 6/7 | | | | 1/7 | | | | | |
| Pop9 | 2 | 1/2 | | | | | | | | | 1/2 |
| Pop10 | 1 | 1/1 | | | | | | | | | |
| Pop11 | 8, 2 | 5/8, 1/2 | 1/8, 1/2 | | 1/8 | | | | 1/8 | | |
| Pop12 | 4 | 3/4 | 1/4 | | | | | | | | |
| Pop13 | 8 | 8/8 | | | | | | | | | |
| Pop14 | 6 | 4/6 | | 1/6 | | | 1/6 | | | | |
| Pop15 | 5 | 5/5 | | | | | | | | | |
| Pop16 | 1 | 1/1 | | | | | | | | | |
| Pop17 | 1 | 1/1 | | | | | | | | | |
| Pop18 | 2 | 2/2 | | | | | | | | | |
| Pop19 | 2 | 2/2 | | | | | | | | | |
| Pop20 | 2 | 2/2 | | | | | | | | | |
